# Periventricular nodular heterotopias is associated with mutation at the *FLNA* locus-a case history and a literature review

**DOI:** 10.1186/s12887-023-04161-4

**Published:** 2023-07-08

**Authors:** Lin Yang, GuangSheng Wu, HuiMei Yin, MengLan Pan, YaFei Zhu

**Affiliations:** Pediatric Department, The Affiliated Hospital of Hangzhou Normal University, No. 126 Wenzhou Road, Hangzhou, Zhejiang 310000 China

**Keywords:** Periventricular nodular heterotopia, *FLNA*, Febrile seizures, Epilepsy

## Abstract

**Background:**

Periventricular nodular heterotopia (PNH), associated with *FLNA* mutations, is a rare clinical condition potentially associated with multiple systemic conditions, including cardiac, pulmonary, skeletal, and cutaneous diseases. However, due to a paucity of information in the literature, accurate prognostic advice cannot be provided to patients with the disease.

**Case presentation:**

We report a 2-year-old female whose PNH was associated with a nonsense mutation in the q28 region of the X chromosome, in exon 31 of *FLNA *(c.5159dupA). The patient is currently seizure-free and has no congenital heart disease, lung disease or skeletal or joint issues, and her development is normal.

**Conclusions:**

*FLNA*-associated PNH is a genetically-heterogeneous disease, and the *FLNA* mutation, c.5159dupA (p.Tyr1720*) is a newly identified pathogenic variant. *FLNA* characterization will help the clinical diagnosis and treatment of PNH and provide individualized genetic counseling for patients.

## Background

*FLNA* is located in the q28 region of the X chromosome [1, 2]. It encodes a widely expressed filamentous protein that acts on intracellular actin binding, and is involved in cell migration, mechano sensing, and cell signaling [3]. *FLNA* variants trigger X-linked filopathies which affect all organs, including the brain, bones, heart, and skin [4]. Periventricular nodular heterotopia (PNH) is strongly associated with *FLNA* mutations, which causes a loss of protein function, meaning developing neuronsfail to differentiate or migrate to the cortex in a timely manner [5]. This causes bilateral gray matter ectopia at the lateral ventricular rim, combined with a large occipital cisterna and hypoplasia of the cerebellum and corpus callosum [1, 6]. As *FLNA* is located on the X chromosome and is prevalent in females, males with PNH may die from severe complications prenatally or at an early age [6]. It is worth noting that the relevance of approximately 1/3 of all *FLNA* mutations is unknown, and that the clinical heterogeneity of *FLNA* mutations is extremely high [7, 8]. Therefore, no genotype-phenotype correlations have been identified [8], which is reflected by a paucity of literature on the subject.

We identified PNH in a 26-month-old girl admitted to our hospital for febrile seizures. The patient’s father had febrile seizures when he was a child, and the patient’s aunt had severe epilepsy and died in infancy, so we did MRI and genetic tests for this patient. Genetic tests identified *FLNA* mutations in exon 31, however, no mutational information in this sub-region was available from the literature. Currently, the girl is not experiencing any epilepsy and no developmental delays. We believe this case report and our literature review could provide individualized treatment plans and better prognoses for patients with *FLNA*-associated PNH disease.

## Case introduction

**Fig. 1:**
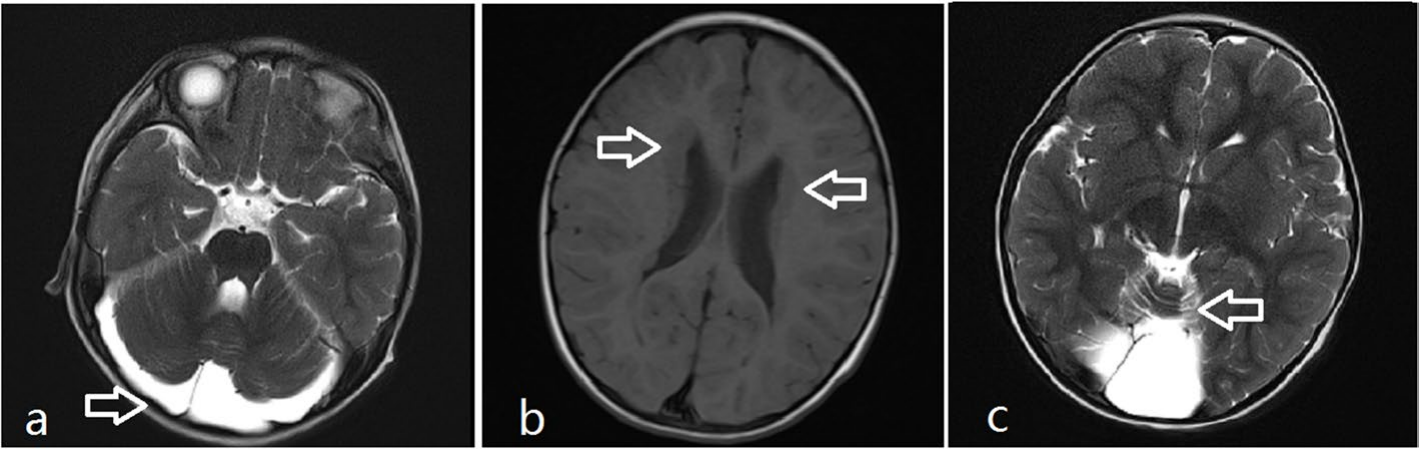
Magnetic resonance imaging (MRI) of the patient suggests multiple nodular gray matter signal shadows in bilateral lateral ventricles, irregular morphology of bilateral lateral ventricles, and significant enlargement of the occipital greater cisterna. The arrow in figure **a** indicates a large occipital cisterna; The arrows in Figure **b** indicate multiple nodular gray matter signals in bilateral lateral ventricles; The arrow in figure **c** indicates hypoplasia of the cerebellum

The female patient was 26 months old. She was admitted to the Department of Pediatrics of the Affiliated Hospital of Hangzhou Normal University in November 2022 with a “2 hour fever and one seizure episode”. The child had one febrile seizure when she was 12 months old, and her aunt (mother’s sister) died of a severe neurological developmental abnormality at a young age. The father had one febrile seizure as a young child. Previously, the girl raised head at 3 months, sat at 8 months, talked at 12 months, walked at 13 months, and currently understood simple vocabulary with no obvious signs of developmental delay. Neurological physical examinations were negative. Post-admission ancillary examinations using photo-stimulated electroencephalogram and 24 h video EEG showed no abnormalities, and no epileptiform discharges were captured. Cardiac ultrasound morphology, structure, and hemodynamics did not show any significant abnormalities. A cranial magnetic resonance imaging (MRI) examination suggested gray matter heterotopia, corpus callosum dysplasia, and an arachnoid cyst in the occipital greater cisterna (Fig. 1).

**Fig. 2:**
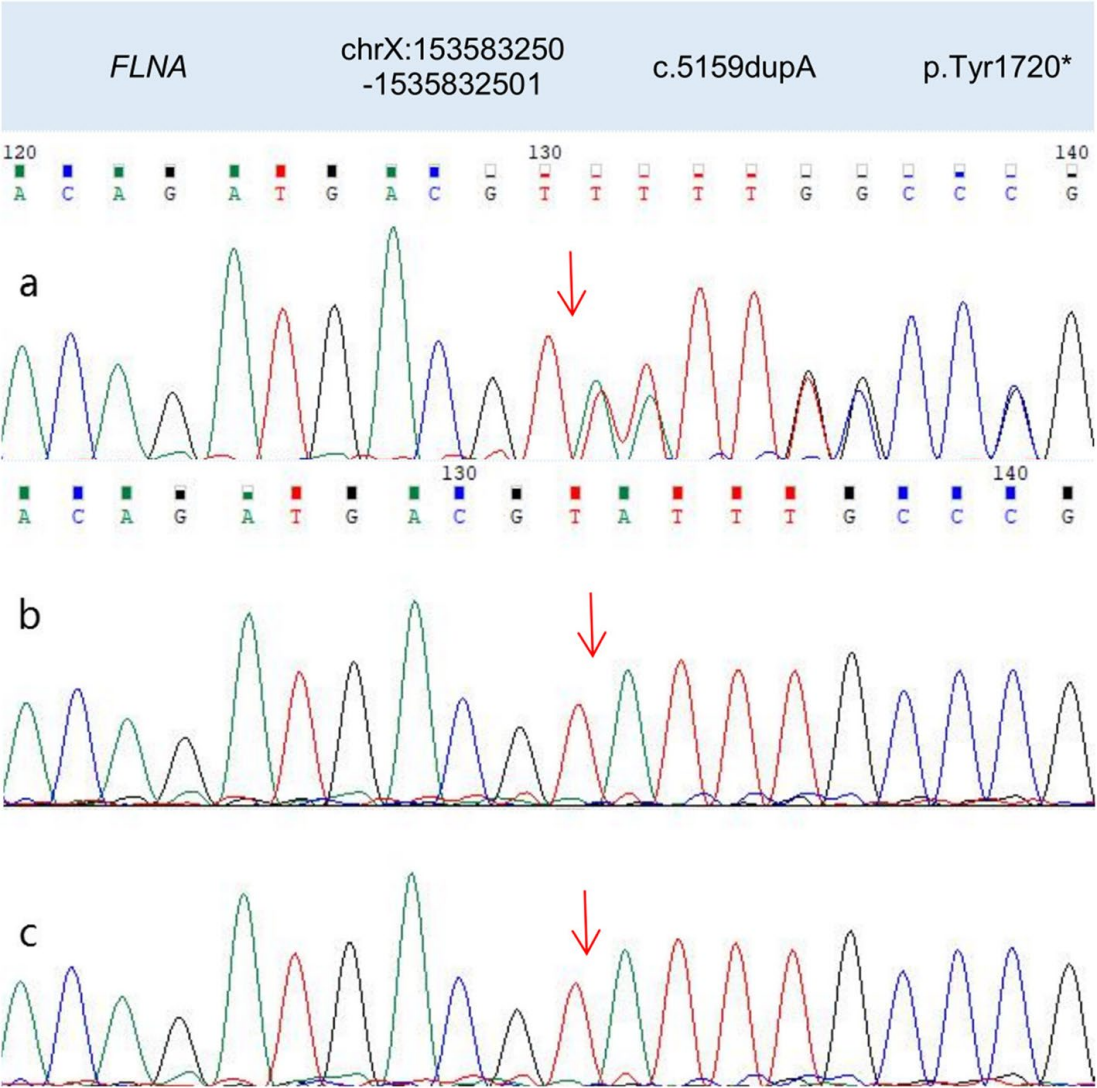
The patient (**a**) has a mutation in exon 31 of *FLNA* (red arrow); a heterozygous nonsense mutation in nucleotide duplication 5159 in the coding region, resulting in protein termination at tyrosine 1720. The patient’s mother and (maternal) grandfather have no mutation information in this region

**Fig. 3:**
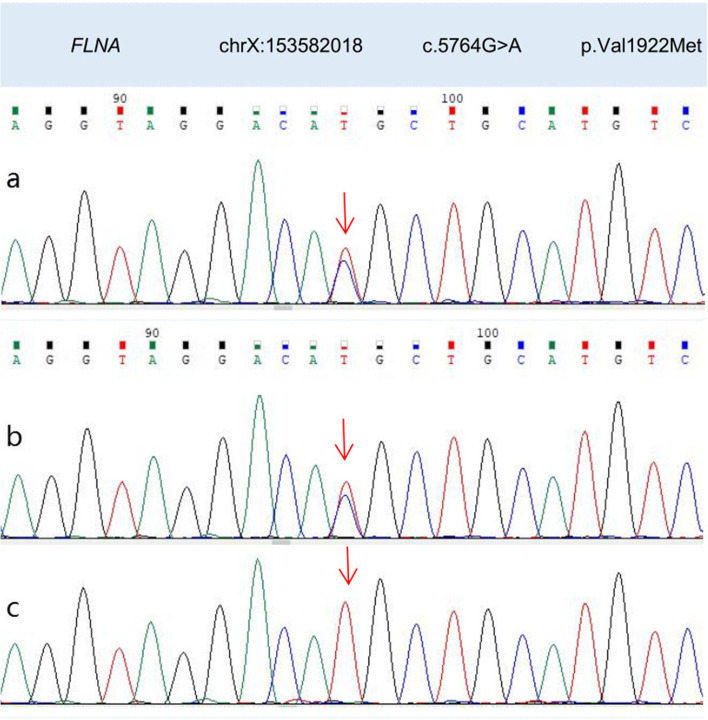
Patient (**a**), patient’s mother (**b**) and patient’s (maternal) grandfather (**c**) have *FLNA* mutationsin exon36 (mutation sites are marked with red arrows). The patient has a heterozygous missense mutation in the coding region at nucleotide 5764 (guanine to adenine), resulting in mutation of amino acid 1922, valine to methionine. The patient’s mother has a heterozygous mutation in c.5764G > A, and the patient’s (maternal) grandfather has a hemizygous mutation in c.5764G > A

**Fig. 4:**
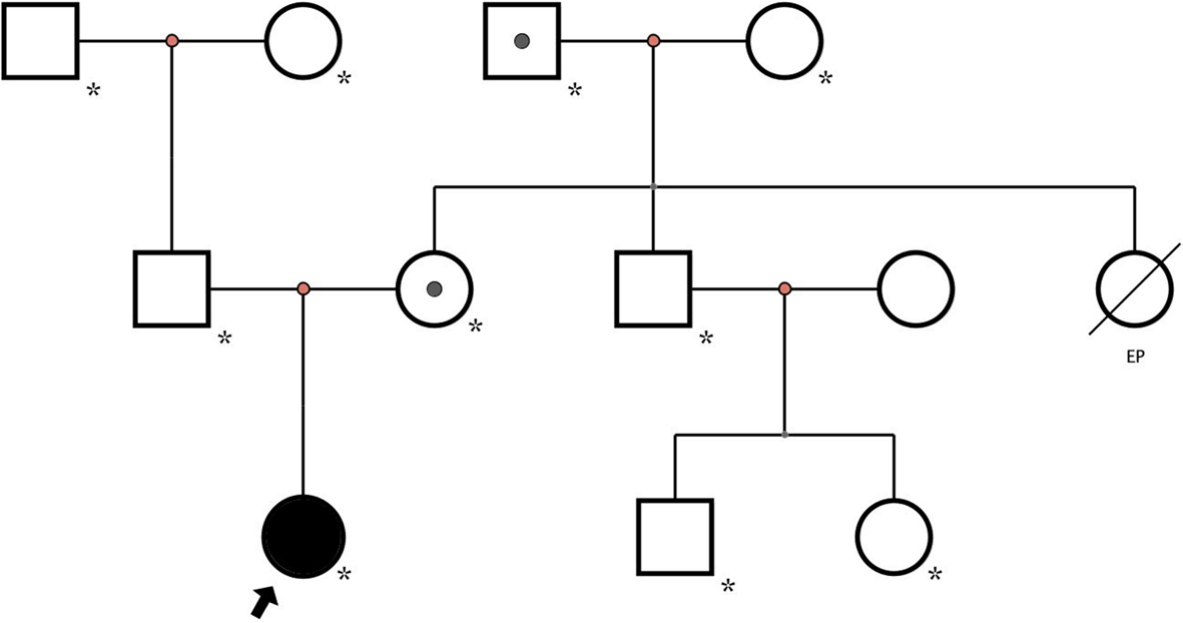
Patient’s family tree. Circle = female. Square = male. Black arrows = index patient. Black dots = *FLNA* with mutations. Slashes = death (this patient had a history of epilepsy). *completed *FLNA* testing in the family

The study was approved by the Medical Ethics Committee of Hangzhou Normal University Hospital (approval number 2021-(E2)-hs-059) in accordance with the ethical guidelines of the Declaration of Helsinki. Written informed consent was obtained from the patient’s guardian. We performed trio whole exon sequencing using targeted region capture high-throughput sequencing, and observed two variants in the patient’s *FLNA* gene. After QC was used to assess the sequencing quality of raw sequencing data and remove low-quality and joint-contaminated reads. The filtered data were sequenced with the human HG19 reference genome using BWA software (Burrows Wheeler Aligner) and the capture effect was assessed. GATK software was used to analyze single nucletide Variant (SNV) and Inde (INSERTION and deletion). 1000 Genomes (1000 Human Genome Dataset), Genome Aggregation Database dataset 2.1.1 and ExAC (The Exome) were used Aggregation Consortium dataset (Aggregation Consortium DATASET) screened the SNV and Indel obtained by analysis. The pathogenicity of false sense mutation and shear mutation was predicted using dbNSFP database. Reported mutations were screened using the Human Mendelian Genetic Database (OMIM), human Gene Mutation Database (HGMD) and Clinvar database. All mutation sites were classified using ACMG genetic variation classification criteria and guidelines. Finally, all possible pathogenic sites were verified by Sanger sequencing. One was in exon31,c.5159dupA:p.Tyr1720* (nucleotide duplication in coding region 5159, resulting in termination at tyrosine 1720) and was considered a heterozygous nonsense mutation. The other occurred at exon36, c.5764G > A:p.Val1922Met (mutation in nucleotide 5764 in the coding region (guanine to adenine), resulting in the mutation of valine (1922) to methionine), and was considered a heterozygous missense mutation (presently not significant). Genetic verification of the patient’s family history revealed a heterozygous mutation in c.5764G > A in the patient’s mother, and a hemizygous mutation in c.5764G > A in the patient’s (maternal) grandfather. Both the mother and (maternal) grandfather underwent cranial MRI, but no PNH was identified; both had no history of epilepsy and were currently healthy. When we processed this information, we hypothesized the child’s PNH was associated with the nonsense mutation in exon 31 of *FLNA *(c.5159dupA). *FLNA* mutations in the patient, the patient’s mother, and the patient’s (maternal) grandfather are shown (Figs. 2 and 3), and the family tree is shown (Fig. 4).

Therefore, the patient had a heterozygous missense mutation in the coding region of *FLNA* at nucleotide 5764 (guanine to adenine), resulting in valine (1922) mutation to methionine. Her mother was heterozygous while her (maternal) grandfather was hemizygous for the mutation.

## Literature review

**Fig. 5:**
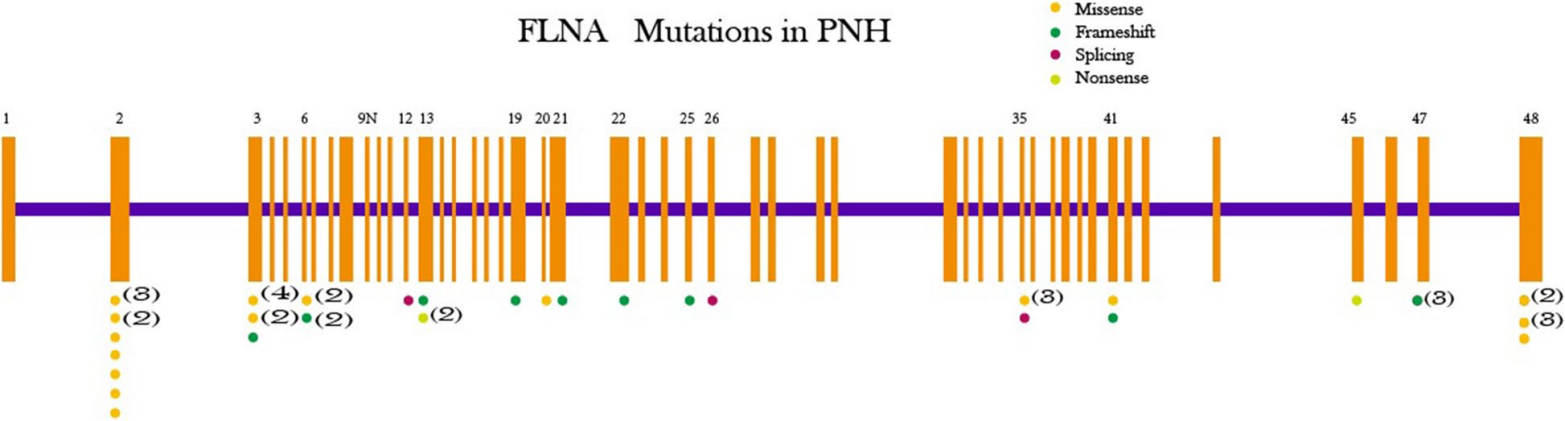
The location of exonic *FLNA* mutations associated with periventricular nodular heterotopia. *FLNA* comprises 49 exons, including a new “poison” exon, 9N. Orange vertical bars represent exons, the purple horizontal line represents introns, numbers above orange bars are exon numbers, numbers in brackets (below orange bars) represent the number of cases included in mutation information, and different color dots represent different *FLNA* mutation types. This is a summary of literature search

We searched PubMed for English-language studies published before February 20th, 2022, using “periventricular nodular heterotopias” AND “FLNA” OR “Grey matter heterotopias” AND “FLNA” terms. We sought studies on PNH associated with *FLNA* mutations, which provided mutation information, such as mutation type in exonic regions, descriptions of clinical symptoms, and images indicating cranial MRI alterations. We retrieved 19 publications covering diverse *FLNA* mutations in PNH patients (Fig. 5). Information on *FLNA* mutations, number of cases, patient gender, MRI imaging descriptions, clinical features, and levels of cognitive development were also gathered (Table 1).

**Table 1:** Periventricular nodular heterotopia associated with *FLNA* mutations – a summary of the literature. F = female, M = male

*FLNA*Mutation information	Exon	Mutation Type	Number of cases	Sex	MRI imaging description	Clinical features	Cognitive development	Refs
c.356T > A	2	missense	1	M	Bilateral lateral ventricular nodule-like gray matter signal, large occipital cisterna	Aortic dilatation with aortic regurgitation and death after severe intraoperative bleeding	Normal	[9]
c.116 C > A	2	missense	1	M	Mild asymmetric nodular gray matter signal in bilateral lateral ventricles, large occipital cisterna	Epilepsy; mucinous degeneration of the heart with mitral valve prolapse	Normal	[9]
c.7778G > T	48	missense	1	F	Isolated gray matter heterotopia in the posterior wall of the right lateral ventricle and isolated gray matter nodule in the frontal horn of the left lateral ventricle	Epilepsy; migraine; rhinorrhea	Normal	[9]
c.622G > C	3	missense	4	F	Bilateral lateral ventricular and ventricular gray matter ectopic nodules	Epilepsy; skeletal dysplasia; Melnick-Needles syndrome	Normal or critical intelligence	[10]
c.4304-1G > A	26	splicing	1	F	Multiple globular nodules located in the lateral ventricles ventricular border	Aortic valve stenosis; interstitial pneumonia; pulmonary hypertension; death at 3 months of age	Not mentioned	[8]
c.7315 C→A	45	splicing	1	F	Bilateral lateral ventricular nodal heterotopia	Dysplasia of the frontal epiphysis.	Normal	[11]
c.987G→C	6	missense	2	F	Bilateral periventricular nodal heterotopia	Refractory epilepsy	Normal	[12]
C.7778G > A	48	missense	2	2 M	Small amount of unilateral lateral ventricular nodular gray matter heterotopia	Epilepsy; one case of left-sided hearing loss; migraine; retinopathy; joint hypermobility; high arched epiglottis	Normal	[13]
c.1923 C > T	13	frameshift	1	M	Lateral ventricular gray matter heterotopia on both sides	Severe constipation; ventricular septal defect, pulmonary artery prolapse and tricuspid valve dysplasia; cerebrofrontal syndrome	Not mentioned	[14]
c.7941_7942delCT	48	missense	3	M	Lateral ventricular gray matter heterotopia; posterior cerebellar cyst	Motor developmental delay; constipation; pseudo-intestinal obstruction; cardiovascular malformation; frontal-facial malformation	Not mentioned	[15]
c.2002 C > T	13	nonsense	2	F	Lateral ventricular gray matter heterotopia on both sides	Thrombocytopenia; mild dilatation of the aortic root with mild aortic regurgitation	Normal	[16]
c.5686G > A	35	missense	3	2F1M	Bilateral lateral periventricular nodal heterotopia; cerebellar giant occipital cisterna	Epilepsy; 1 female with mental retardation; 1 female with patent ductus arteriosus	Behind or normal	[17]
c.245 A > T	2	missense	3	F	Bilateral lateral periventricular nodal heterotopia; cerebellar giant occipital cisterna	Two women with epilepsy	Critical or normal	[18]
c.7627_7634del	47	frameshift	3	F	Bilateral lateral periventricular nodal heterotopia; cerebellar giant occipital cisterna	Twowomen with epilepsy	Critical or normal	[18]
c.220G > A	2	missense	1	F	Periventricular nodular ectopia	Recurrent respiratory infections, bilateral pulmonary atelectasis, pulmonary cysts, bronchial softening, pulmonary hypertension, asthma and chronic oxygen dependence; secondary atrial septal defect, aortic constriction and mild aortic valve closure insufficiency; motor retardation; hypotonia and excessive joint laxity	Normal	[19]
c.5683G→T	35	splicing	1	F	Bilateral ventricular nodal heterotopia; delayed myelin formation; enlarged subarachnoid space	Preterm delivery at 30 weeks; cystic lung lesion; pulmonary hypertension	Lagging behind	[20]
c.6769G > C	41	missense	1	M	Diffuse periventricular gray matter heterotopia	Adolescent distal upper extremity muscular dystrophy; joint hypermobility syndrome	Normal	[21]
c.883_890 8 bp deficiency	6	frameshift	2	F	Bilateral periventricular gray matter nodules	Dyslexia; a woman with an aortic aneurysm	Normal	[22]
c.4147delG	25	frameshift	1	F	Bilateral ventricular nodal heterotopia	aortic aneurysm;joint hypermobility	Normal	[23]
c.2762delG	19	frameshift	1	F	Bilateral ventricular nodal heterotopia	mitral and aortic valves with mucus-like changes,mild regurgitation; joint hypermobility	Normal	[23]
c.C116G→A39G	2	missense	1	F	Bilateral ventricular nodal heterotopia	Epilepsy; aortic aneurysm; joint hypermobility	Mildly behind	[23]
c304A > G	2	missense	2	1F1M	Bilateral ventricular nodal heterotopia;Cerebellar hypoplasia	One male with cryptorchidism and patent ductus arteriosus	Normal but low	[24]
c446C > T	3	missense	2	1M1F	M left lateral ventricular isolated nodal heterotopia; F right lateral ventricular continuum gray matter heterotopia	Epilepsy; aortic valve closure insufficiency	Normal but low	[24]
c.568_569insG	3	frameshift	1	F	Bilateral ventricular nodal heterotopia	Epilepsy	Normal	[25]
c.1692_2A > G	12	splicing	1	M	Bilateral ventricular nodal heterotopia;Cerebellar hypoplasia	Epilepsy; aortic aneurysm	Critical level	[25]
c.3035 C > T	20	missense	1	M	Bilateral ventricular nodal heterotopia;Wide ventricles; double splitting of the pellucid septum, etc.	Cardiac malformations, including single atrium, mitral atresia, left ventricular hypoplasia, etc.	Not mentioned	[26]
c.220G > A	2	missense	1	F	Bilateral ventricular nodal heterotopia;Retrocerebellar cysts	Aortic constriction; excessive skin joint laxity; frontal facial deformity; congenital lobar emphysema with bronchial tenderness	Mild retardation	[26]
c.3045del5	21	frameshift	1	F	Bilateral ventricular nodal heterotopia	Aortic closure insufficiency; pararenal aortic aneurysm.	Normal	[26]
c.3582delC	22	frameshift	1	F	Bilateral ventricular nodal heterotopia	Epilepsy; mild aortic stenosis with regurgitation	Normal	[26]
c.6635delTCAG	41	frameshift	1	F	Bilateral ventricular nodal heterotopia	Ventricular septal defect, aortic closure insufficiency; migraine attacks with aphasia	Not mentioned	[26]

## Discussion

In previous PNH cases, patients were shown to have refractory epilepsy, cognitive and developmental impairment, and were mostly associated with a poor prognosis [5]. In our patient, we confirmed PNH was associated with *FLNA*, consistent with other *FLNA*-associated mutations in other patients with PNH and their family lines. However, with advanced precision medicine, diseases associated with *FLNA* mutations are now reported more frequently, with an increasing emphasis on genetic heterogeneity [6, 7, 27].

The diseases associated with *FLNA* mutation are known as X-linked filopathies due to the critical role of *FLNA* in organ development in humans [4]. PNH linked-*FLNA* mutations are associated with cardiovascular disease, malformations in the frontal face, congenital lung disease, excessive laxity of the skin and joints, and platelet abnormalities [28–30]. We observed a definite female prevalence for PNH associated-*FLNA* mutations, however, their overall prognosis was superior to males [31]. Moreover, in a larger number of cases, many patients were cognitively normal and had completed their university studies [32]. More interestingly, we showed that the proportion of febrile seizures was higher in patients with PNH [33, 34], consistent with our case who experienced these seizures and was subsequently diagnosed with PNH. However, associations between *FLNA* mutations and febrile seizures remain to be fully investigated.

From the literature, it was suggested that *FLNA* mutation type and exonic region could be correlated with clinical prognosis [13, 35, 36]. In males, survival and phenotype disease severity associated with missense mutations and distal truncation mutations were relatively positive, however, phenotypes associated with gene fragment insertions and deletions could be fatal [7]. From the literature, exon 2 mutations were the most reported; all were missense, with variable patient prognoses. Therefore, from the limited available information on *FLNA*-associated PNH, we hypothesize the mutation type and associated exonic region are not predictive of a clinical prognosis.

Our patient had no PNH family history, no current epilepsy, and cognitive and motor development was normal. Genetic characterization of the family showed the (maternal) grandfather and mother had a c.5764G > A mutation in exon 36 of *FLNA*, but MRIs showed no ectopic changes in their ventricular gray matter. For patients with a family history of febrile seizure and epilepsy, it is necessary to improve cranial MRI and genetic testing at the time of the first febrile seizure, which can lead to early diagnosis and prognosis. It was worth noting, in the literature, we observed no exon 36 *FLNA* mutation associations with PNH. When combined with our family’s genetic profile, we believe the missense mutation in this exon is not associated with PNH in our patient, but the c.5159dupA nonsense mutation in exon 31 may be the cause of her PNH. Importantly, this is the first PNH-associated case study in this exon in the literature. Based on patient clinical examinations, severe cardiac disease, pulmonary disease, and excessive skin and joint laxity have been ruled out, which suggests a good prognosis for this patient.

## Conclusions

In clinical settings, PNH is a rare neuro developmental disease, therefore *FLNA* variants should be clarified as soon as possible after a PNH diagnosis. *FLNA* variants can cause X-linked filopathies, which potentially affect several important organs [32]. We reported a female child with PNH whose disease was associated with a nonsense mutation in exon31 of *FLNA* in the q28 region of the X chromosome. Currently, the patient is developing normally, with no seizures, and no congenital heart disease, lung disease, or skeletal and joint issues.

Diseases associated with *FLNA* variants are genetically heterogeneous, therefore, early and comprehensive clinical evaluations could help patient survival and social functioning in later life.

## Data Availability

All data generated or analysed during this study are included in this published article.

## Abbreviations

PNH: Periventricular nodular heterotopia
*FLNA*: Filamin A
